# Interactive rather than independent effect of *APOE* and sex potentiates tau deposition in women

**DOI:** 10.1093/braincomms/fcab126

**Published:** 2021-06-07

**Authors:** Yi-Ting T Wang, Tharick A Pascoal, Joseph Therriault, Min Su Kang, Andréa L Benedet, Melissa Savard, Cécile Tissot, Firoza Z Lussier, Jaime Fernandez Arias, Sulantha Mathotaarachchi, Maria Natasha Rajah, Serge Gauthier, Pedro Rosa-Neto, for the Alzheimer’s Disease Neuroimaging Initiative

**Affiliations:** Translational Neuroimaging Laboratory, The McGill University Research Centre for Studies in Aging, McGill University, Montréal, QC, Canada; Douglas Research Centre, Montréal, QC, Canada; Department of Psychiatry, University of Pittsburgh, Pittsburgh, PA, USA; Translational Neuroimaging Laboratory, The McGill University Research Centre for Studies in Aging, McGill University, Montréal, QC, Canada; Douglas Research Centre, Montréal, QC, Canada; Translational Neuroimaging Laboratory, The McGill University Research Centre for Studies in Aging, McGill University, Montréal, QC, Canada; Douglas Research Centre, Montréal, QC, Canada; Translational Neuroimaging Laboratory, The McGill University Research Centre for Studies in Aging, McGill University, Montréal, QC, Canada; Translational Neuroimaging Laboratory, The McGill University Research Centre for Studies in Aging, McGill University, Montréal, QC, Canada; Translational Neuroimaging Laboratory, The McGill University Research Centre for Studies in Aging, McGill University, Montréal, QC, Canada; Douglas Research Centre, Montréal, QC, Canada; Translational Neuroimaging Laboratory, The McGill University Research Centre for Studies in Aging, McGill University, Montréal, QC, Canada; Douglas Research Centre, Montréal, QC, Canada; Translational Neuroimaging Laboratory, The McGill University Research Centre for Studies in Aging, McGill University, Montréal, QC, Canada; Douglas Research Centre, Montréal, QC, Canada; Translational Neuroimaging Laboratory, The McGill University Research Centre for Studies in Aging, McGill University, Montréal, QC, Canada; Douglas Research Centre, Montréal, QC, Canada; Department of Psychiatry, McGill University, Montréal, QC, Canada; Translational Neuroimaging Laboratory, The McGill University Research Centre for Studies in Aging, McGill University, Montréal, QC, Canada; Douglas Research Centre, Montréal, QC, Canada; Department of Psychiatry, McGill University, Montréal, QC, Canada; Department of Neurology and Neurosurgery, McGill University, Montréal, QC, Canada; Translational Neuroimaging Laboratory, The McGill University Research Centre for Studies in Aging, McGill University, Montréal, QC, Canada; Douglas Research Centre, Montréal, QC, Canada; Department of Psychiatry, McGill University, Montréal, QC, Canada; Department of Neurology and Neurosurgery, McGill University, Montréal, QC, Canada

**Keywords:** *APOE*, positron emission tomography, sex difference, tau, Alzheimer’s disease

## Abstract

The apolipoprotein E gene (*APOE*) is the most important genetic risk factor for sporadic Alzheimer disease, with the *ε4* allele being associated with increased cerebral amyloid-β and tau pathologies. Although *APOE* has been suggested to have a stronger effect in women as compared to men, there is a lack of comprehensive assessment on how the interactive effect of *APOE* and sex modulates regional vulnerability to tau accumulation. We previously have shown the regional vulnerability to the interactive effect of tau and *APOE*, yet the sex difference was not specifically addressed. In this study, we leveraged PET imaging data from the Translational Biomarkers in Aging and Dementia cohort at McGill University Research Centre for Studies in Aging to elucidate the *APOE-*by-sex interactive effect on tau burden. We hypothesized sex-dependent regional vulnerability to tau deposition. PET radiopharmaceuticals [^18^F]AZD4694 and [^18^F]MK6240 were used to assess amyloid-β and tau level respectively in 277 subjects from the Translational Biomarkers in Aging and Dementia cohort. We found that the interaction between *APOE* and sex, rather than their independent main effects, was associated with abnormal tau accumulation in medial temporal regions. Specifically, we found that female *APOEε4* carriers showed significantly higher tau burden in early tau deposition regions including the hippocampus, entorhinal and parahippocampal cortices, after accounting for age, educational attainment, clinical diagnosis and neocortical amyloid load. We replicated these findings in 221 subjects from the Alzheimer’s Disease Neuroimaging Initiative cohort, in which a different tau-PET radioligand, [^18^F]flortaucipir, was used to assess tau burden. In conclusion, this study provides evidence from two cohort studies that interactive rather than independent effect of *APOE* and sex potentiates early tau deposition in women. Our results have important implications for clinical trials and practice, which should take into consideration both *APOEε4* carriage status and sex for identifying individuals with the highest probability of developing tau accumulation and clinical progression.

## Teaser

Wang et al. report that *APOEε4* potentiates tau burden in medial temporal regions including hippocampus, entorhinal and parahippocampal cortices in women, but not in men. The results suggest that *APOE*-by-sex interactive effect, rather than their main effects, modulates regional vulnerability to tau accumulation.

## Graphical Abstract

**Graphical Abstract:**
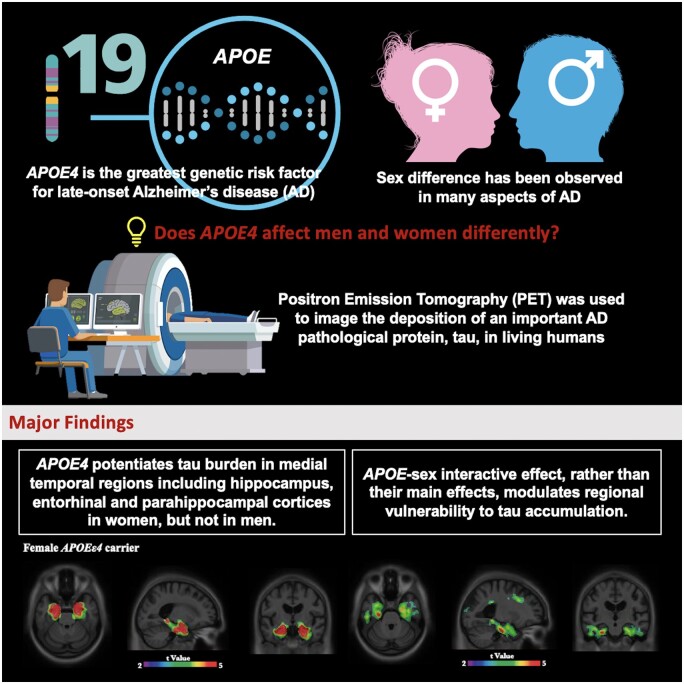

## Introduction

Apolipoprotein E (*APOE*) is the strongest genetic risk factor for sporadic Alzheimer’s disease,^1^ with the ε4 allele conferring increased risk. The ε4 allele increases the risk for dementia due to Alzheimer’s disease in a dose-dependent manner, in which the presence of one ε4 allele is linked with earlier development of Alzheimer’s disease,^2^ and homozygosity of *APOEε4* is associated with the onset of Alzheimer’s disease 10 years earlier compared with non-ε4 carriers.^3^*APOE* has been implicated in various neuropathological cascades relevant to Alzheimer’s disease, including alterations in cerebral glucose metabolism,^4–6^ amyloidosis,^7–9^ tau tangle pathology,^10–12^ microglial activation^13^^,^^14^ and neurodegeneration.^11^^,^^15^ Large meta-analysis showed a stronger association between *APOE* and CSF tau levels among females in comparison to males.^16^ In addition, a recent study demonstrated that in cognitively normal older adults, women had more tau tangles in the entorhinal cortex than did men, and this sex difference was slightly more pronounced in *APOEε4* carriers.^17^ Indeed, there is a growing emphasis that highlights sex as an essential consideration in Alzheimer’s disease models to move the field towards more effective prevention and treatment strategies.^18^^,^^19^

The role that sex plays in Alzheimer’s disease has long been the subject of intense investigation.^20^^,^^21^ A considerable amount of literature suggests that there is a disproportionally high prevalence of Alzheimer’s disease in women.^22–25^ Beside sex-specific patterns of clinical manifestation,^26^ sex also affects the rates of cognitive decline, cerebral atrophy, as well as the response to treatments.^27–32^ Previous works reported conflicting results about the sex-specific effect of *APOEε4* on tau,^33–35^ and thus a comprehensive assessment on how *APOE* and sex modulates tau pathology is of critical importance. As we demonstrated a regional-dependent association between *APOEε4* and Alzheimer’s disease pathophysiology,^12^^,^^36^ we hypothesized that an *APOE*-by-sex interactive effect modulates regional vulnerability to tau accumulation across the spectrum of clinical manifestations of Alzheimer’s disease, such that women with an *APOEε4* genotype exhibit greater accumulation of tau in the medial temporal lobes. The overarching goal of this study was to investigate the effect of *APOE*-by-sex interaction on brain tau burden.

## Materials and methods

### Participants

#### Translational biomarkers in ageing and dementia

The Translational Biomarkers in Aging and Dementia (TRIAD) cohort aims at describing biomarker trajectories and interactions as drivers of dementia. The TRIAD study was launched in 2017 as part of the McGill Centre for Studies in Aging. In this study, a total of 277 subjects were assessed, among which 40 were diagnosed with dementia due to Alzheimer’s disease and 60 with mild cognitive impairment (MCI); 142 cognitively unimpaired individuals and 35 young healthy controls were also recruited. All participants underwent structural MRI, amyloid-PET with [^18^F]AZD4694, tau-PET with [^18^F]MK6240 and genotyping for *APOEε4*. They also had detailed clinical assessments including Mini-Mental State Examination and Clinical Dementia Rating (CDR). Cognitively unimpaired individuals had a CDR of zero. Participants with MCI had a CDR of 0.5, subjective and objective memory impairments and essentially normal activities of daily living. Participants with dementia due to Alzheimer’s disease had a CDR between one and two, in addition to meeting the National Institute on Aging and the Alzheimer’s Association criteria for probable Alzheimer’s disease. Diagnosis of participants was confirmed by an expert consensus panel of clinicians, neuropsychologists and nurses, based on both neuropsychological and neuroimaging data (including MRI, amyloid-PET and tau-PET). Similar to other longitudinal cohort studies of ageing and Alzheimer’s disease, the TRIAD cohort is enriched for *APOEε4* carriers. Inclusion criteria for all participants are the ability to speak English or French, good general health (no diseases expected to interfere with study participation over time), absence of claustrophobia, and adequate visual and auditory capacities to follow neuropsychologic evaluation. This study’s protocol was approved by Douglas Mental Health institutional review board and informed written consent was obtained from each participant.

#### Alzheimer’s Disease Neuroimaging Initiative

In this study, we assessed Alzheimer’s disease patients (*n* = 21), MCI patients (*n* = 81) and cognitively unimpaired individuals (*n* = 119) from the Alzheimer’s Disease Neuroimaging Initiative (ADNI) cohort. The participants all underwent structural MRI, amyloid-PET with [^18^F]florbetapir, tau-PET with [^18^F]flortaucipir and genotyping for *APOEε4*. Cognitively unimpaired individuals had a CDR of zero, participants with MCI had a CDR of 0.5 and participants with Alzheimer’s disease had a CDR of one or greater in addition to meeting standard diagnostic criteria.^37^ Data used in the preparation of this article were obtained from the ADNI database. The ADNI was launched in 2003 as a public–private partnership, led by Principal Investigator Michael W. Weiner, MD. The primary goal of ADNI has been to test whether serial MRI, PET, other biological markers, and clinical and neuropsychological assessment can be combined to measure the progression of MCI and early Alzheimer’s disease. The ADNI study was approved by the institutional review boards of all of the participating institutions. Informed written consent was obtained from all participants at each site. Full information regarding the inclusion and exclusion criteria in ADNI can be accessed at http://adni.loni.usc.edu/. There was no attempt to match cases between the two study cohorts.

### Genetic analyses

#### Translational Biomarkers in Aging and Dementia

*APOE* genotyping was done for participants recruited at McGill Centre for Studies in Aging using DNA from blood samples. Determination of *APOE* genotypes was carried out by polymerase chain reaction amplification, followed by restriction enzyme digestion, and subsequent standard gel resolution and visualization processes. All *APOE* genotype data underwent further QC checks, including sex and identity checks. Full details of this procedure can be found elsewhere.^38^

#### Alzheimer’s Disease Neuroimaging Initiative

Determination of *APOE* genotypes for ADNI patients took place at the University of Pennsylvania Alzheimer Disease Biomarker Laboratory. Complete details of genetic methods used in ADNI can be accessed at http://adni.loni.usc.edu/data-samples/clinical-data/.

### Positron emission tomography image acquisition and processing

#### Translational Biomarkers in Aging and Dementia

**Figure 1:**
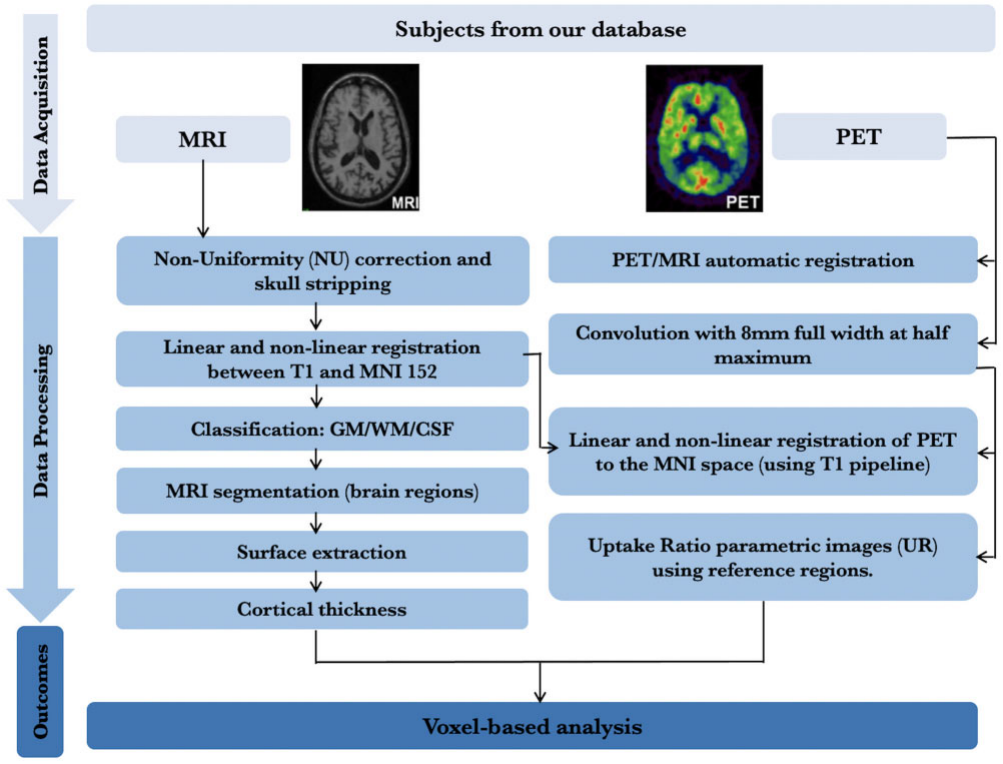
**Imaging analytical pipeline.** *MRI:* T_1_-weighted images were non-uniformity and field-distortion corrected, followed by PET images automatically registered to the T_1_-weighted image space. T_1_-weighted images were then linearly and non-linearly registered to the MNI reference space, and further be performed with classification (classified into grey matter, white matter and cerebrospinal fluid), segmentation and surface extraction. Cortical thickness was also calculated. *PET:* PET images were spatially smoothed to achieve a final resolution of 8 mm full width at half maximum. Subsequently, they were registered to the MNI space using the transformations from the T_1_-weighted image to MNI space and from the PET image to T_1_-weighted image space. [^18^F]AZD4694 PET SUVR maps were generated using the cerebellar grey matter as the reference region, and [^18^F]MK6240 PET SUVR maps were generated using the inferior cerebellar grey matter as the reference region.

All individuals had a 3D T_1_-weighted MRI (3T Siemens) and [^18^F]AZD4694 and [^18^F]MK6240 PET scans acquired with a brain-dedicated Siemens High-Resolution Research Tomograph in the Montreal Neurological Institute. The [^18^F]AZD4694 ([^18^F]NAV4694) scans were acquired 40–70 min after the intravenous bolus injection of the tracer, and scans were reconstructed with the ordered subset expectation maximization algorithm on a four-dimensional volume with 3 frames (3 × 600 s).^39^ The [^18^F]MK6240 images were acquired 90–110 min post-injection, and scans were reconstructed with the ordered subset expectation maximization algorithm on a four-dimensional volume with 4 frames (4 × 300 s).^40^ At the end of each PET acquisition, a 6-min transmission scan was conducted with a rotating ^137^Cs point source for attenuation correction. The images were additionally corrected for motion, dead time, decay, and random and scattered coincidences. The detailed imaging analytical pipeline is illustrated in Fig. 1. Briefly, T_1_-weighted images were non-uniformity and field-distortion corrected. PET images were then automatically registered to the T_1_-weighted image space, and the T_1_-weighted images were linearly and non-linearly registered to the MNI reference space.^41^ The PET images were spatially smoothed to achieve a final resolution of 8 mm full width at half maximum. Subsequently, PET images were meninges and skull stripped, and linearly and non-linearly registered to the MNI space using the transformations from the T_1_-weighted image to MNI space and from the PET image to T_1_-weighted image space. The [^18^F]AZD4694 standardized uptake value ratio (SUVR) maps were generated using the cerebellar grey matter as the reference region,^39^ and the [^18^F]MK6240 SUVR maps were generated using the inferior cerebellar grey matter as the reference region.^40^ A global [^18^F]AZD4694 SUVR value was estimated for each participant by averaging the SUVR from the precuneus, prefrontal, orbitofrontal, parietal, temporal, anterior and posterior cingulate cortices.^42^^,^^43^ Regional [^18^F]MK6240 SUVRs were generated for each Braak staging regions of interest (ROI) and meta ROIs. Braak staging ROIs include transentorhinal regions (I–II), limbic regions (III–IV) and isocortical association regions (V–VI). Meta ROIs include entorhinal, amygdala, parahippocampal, fusiform, inferior temporal and medial temporal regions.

#### Alzheimer’s Disease Neuroimaging Initiative

Full information regarding acquisition and pre-processing of PET data in ADNI is provided at http://adni.loni.usc.edu/data-samples/pet/. Pre-processed PET images downloaded from ADNI underwent spatial normalization to the ADNI standardized space using the transformations of PET native to MRI native space and MRI native to the ADNI space. [^18^F]flortaucipir (also known as [^18^F]T807 and/or [^18^F]AV1451) SUVR maps were generated using the inferior cerebellar grey matter as a reference region,^44^ and [^18^F]florbetapir SUVR maps were generated using the cerebellar grey matter as a reference region. A global [^18^F]florbetapir SUVR value was estimated for each participant by averaging the SUVR from the precuneus, prefrontal, orbitofrontal, parietal, temporal, anterior and posterior cingulate cortices.^42^ Regional [^18^F]flortaucipir SUVRs were generated for each Braak staging ROI as well as meta ROIs.

### Statistical analyses

Data from two independent cohorts TRIAD and ADNI were investigated. The primary outcome measure of the study was tau load measured by tau-PET using [^18^F]MK6240 (TRIAD) and [^18^F]flortaucipir (ADNI).

Demographic and clinical data were assessed using *t*-test, Tukey’s *post hoc* analysis and contingency chi-square tests. Multivariate linear regression models were performed in MATLAB R2015a (The MathWorks, Natick, MA, USA) to evaluate the *APOE* main effect, the sex main effect, as well as the *APOE-*by-sex interaction on tau burden in the Braak staging ROIs and meta ROIs. Braak staging ROIs include transentorhinal regions (I–II), limbic regions (III–IV) and isocortical association regions (V–VI). Meta ROIs include entorhinal, amygdala, parahippocampal, fusiform, inferior temporal and medial temporal regions. To ensure that the results were not driven by the effect of clinical status, we adjusted the models for clinical diagnosis. The models were also corrected for age and education level. Because *APOEε4* is associated with amyloid-PET uptake, the neocortical Aβ SUVR was also used as a covariate in the models.

#### Voxelwise analyses

Neuroimaging voxel-based analyses were performed using VoxelStats toolbox to investigate the relationships between sex, *APOEε4* carriage status and tau burden. VoxelStats toolbox (https://github.com/sulantha2006/VoxelStats) is a MATLAB-based analytical framework that allows for the execution of multimodal voxelwise neuroimaging analyses. The voxel-based models outlined below were built to test whether *APOE* main effect, sex main effect and *APOE-*by-sex interactive effect is associated with tau level. In every brain voxel, the model was of the form:

Independent model:

Tau burden=βo+β1(APOE)+β2(sex)+covariates+ε

and

 Interactive model:

Tau burden=βo+β1(APOE)+β2(sex)+β3(APOE*sex)+covariates+ε.

T-statistical parametric maps were corrected for multiple comparisons using a random field theory cluster threshold of *P *<* *0.001, overlaid on the Alzheimer's Disease Neuroimaging Initiative reference template. Tau/amyloid ratiomaps were created by dividing tau-PET uptake with amyloid-PET uptake in every voxel to account for amyloid load. Age, education level and clinical diagnosis were used as covariates in the models.

### Data availability

Data used in the preparation of this article were obtained from the ADNI database. Full information regarding the ADNI inclusion and exclusion criteria, complete details of genetic methods used in ADNI, and information regarding acquisition and pre-processing of PET data can be accessed at http://adni.loni.usc.edu/. Data of the TRIAD cohort that support the findings of this study are available from the corresponding author upon reasonable request.

## Results

We studied 277 individuals from the TRIAD cohort (35 cognitively unimpaired young, 142 cognitively unimpaired elderly, 60 MCI and 40 Alzheimer’s disease dementia), and 221 individuals from the ADNI cohort (119 cognitively unimpaired elderly, 81 MCI and 21 Alzheimer’s disease dementia). Participant demographics and clinical information are summarized in Table 1.

**Table 1:** Demographics of study populations

		TRIAD			ADNI		
	Young	CU	MCI	AD	CU	MCI	AD
No.	35 (12.6%)	142 (51.3%)	60 (21.7%)	40 (14.4%)	119 (53.8%)	81 (36.6%)	21 (9.5%)
Sex (male: female)	13:22	49:93	29:31	19:21	59:60	43:38	9:12
Age, mean (SD), year	22.8 (1.8)	69.1 (10.2)^a^	72.0 (6.9)^a^	66.7 (8.0)^a, c^	77.7 (6.4)	77.2 (7.1)	82.3 (6.8)^e, f^
Education, mean (SD), year	16.5 (1.5)	15.4 (3.7)	14.6 (3.5)^a^	14.8 (3.4)	16.6 (2.6)	16.1 (2.9)	15.1 (2.5)^e^
MMSE score, mean (SD)	29.8 (0.4)	29.1 (1.0)	27.8 (1.9)^a, b^	19.3 (7.2)^a, b, c^	29.0 (1.3)	28.3 (1.8)	22.1 (5.2)^e, f^
*APOE* ε*4* carriage status	22.8%	29.6%	41.7%	60.0%^a, b^	30.0%	37.2%	50.0%
Tau-PET SUVR, mean (SD)							
Braak I	0.88 (0.13)	1.07 (0.32)	1.73 (0.91)^a, b^	2.54 (0.87)^a, b, c^	1.08 (0.11)	1.16 (0.17)^e^	1.29 (0.24)^e, f^
Braak II	0.70 (0.11)	0.89 (0.15)^a^	1.12 (0.38)^a, b^	1.41 (0.36)^a, b, c^	1.25 (0.14)	1.29 (0.15)	1.32 (0.15)
Braak III	0.98 (0.08)	1.03 (0.14)	1.27 (0.47)^a, b^	2.53 (1.06)^a, b, c^	1.12 (0.11)	1.18 (0.15)^e^	1.30 (0.24)^e, f^
Braak IV	1.03 (0.10)	1.06 (0.12)	1.33 (0.49)^a, b^	2.76 (1.14)^a, b, c^	1.12 (0.14)	1.18 (0.15)^e^	1.28 (0.29)^e, f^
Braak V	1.13 (0.13)	1.06 (0.13)	1.21 (0.30)	2.53 (1.19)^a, b, c^	1.01 (0.12)	1.06 (0.14)^e^	1.10 (0.21)^e^
Braak VI	1.11 (0.12)	1.04 (0.11)	1.10 (0.16)	1.85 (0.86)^a, b, c^	0.97 (0.09)	1.00 (0.07)^e^	1.02 (0.15)^e^
Meta ROI	1.04 (0.11)	1.08 (0.15)	1.40 (0.53)^a, b^	2.69 (1.01)^a, b, c^	1.15 (0.09)	1.21 (0.16)^e^	1.43 (0.40)^e, f^

Significant differences indicate results of the analysis of variance to assess the difference between groups except for sex and APOE ε*4* status, where a contingency chi-square was performed. Tukey’s *post hoc* analysis tested significant differences from (TRIAD) ^a^Young; ^b^CU; ^c^MCI; ^d^AD; and (ADNI) ^e^CU; ^f^MCI; ^g^AD. AD = Alzheimer’s disease; MCI = mild cognitive impairment; CU = cognitively unimpaired; Young, young cognitively normal; MMSE = Mini-Mental State Examination.

### Sex, *APOE* and Tau load

**Figure 2:**
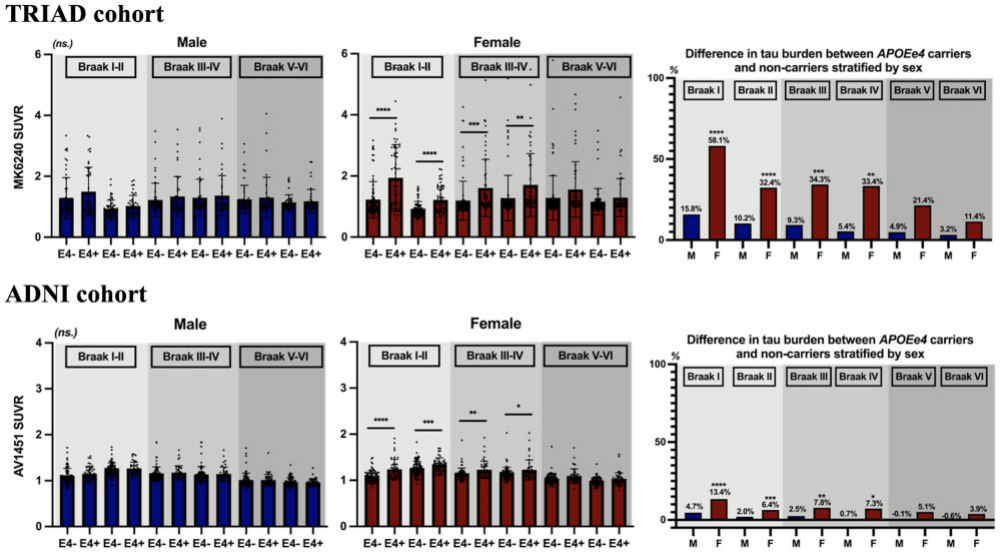
**Female *APOE*ε4 carriers showed higher tau load in Braak staging I–IV ROIs.** *TRIAD: Male:* No significant effect of *APOE* on tau burden was observed between *APOE*ε4 carriers and *APOE*ε4 non-carriers. *Female: APOE*ε4 carriers showed 58.1%, 32.4%, 34.3% and 33.4% higher [^18^F]MK6240 SUVR in Braak staging I–IV ROIs, respectively, as compared to *APOE*ε4 non-carriers. *ADNI: Male:* No significant effect of *APOE* on tau burden was observed between *APOE*ε4 carriers and *APOE*ε4 non-carriers. *Female:* Tau levels in Braak staging I–IV ROIs were significantly higher in *APOE*ε4 carriers in comparison to *APOE*ε4 non-carriers. ****P < 0.001, ***P < 0.005, **P < 0.01, *P < 0.05. Independent samples *t*-tests were performed for comparisons between *APOE*ε4 carriers and *APOE*ε4 non-carriers regarding their tau burden in different ROIs.

**Figure 3:**
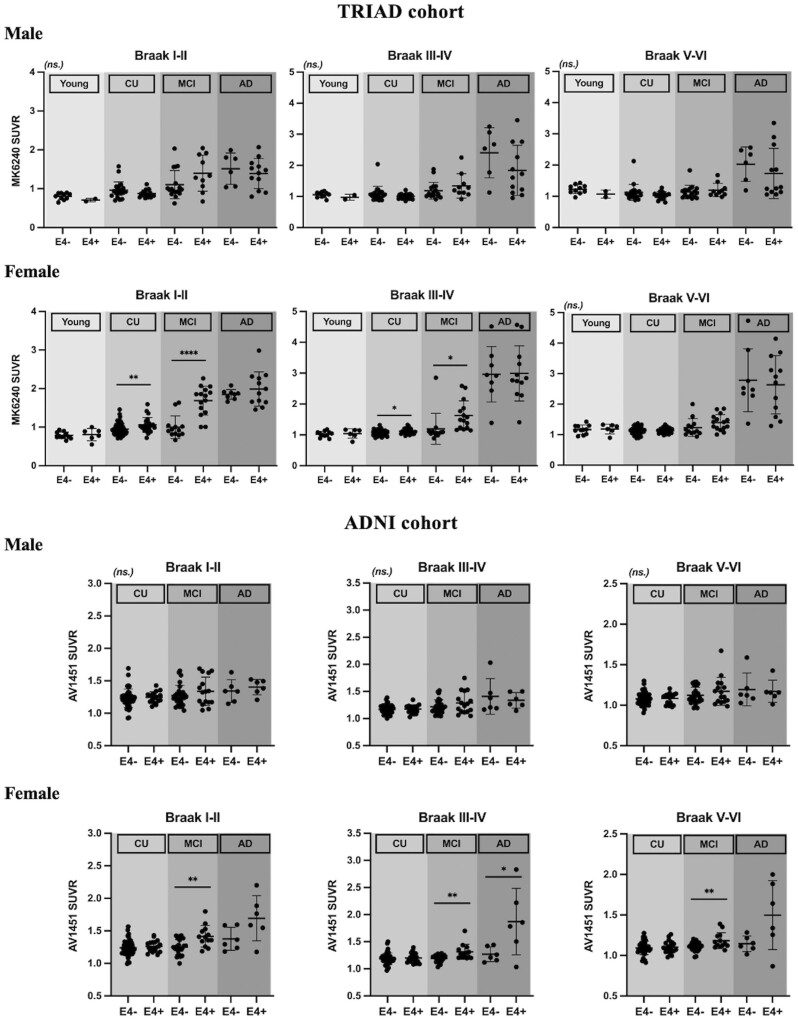
**Tau-PET SUVRs were elevated in female *APOE*ε4 carriers with mild cognitive impairment in comparison to *APOE*ε4 non-carriers.** *TRIAD: Male:* No significant difference in [^18^F]MK6240 SUVRs was observed between *APOE*ε4 carriers and *APOE*ε4 non-carriers. *Female:* Both cognitively unimpaired and mild cognitively impaired *APOE*ε4 carriers showed significantly higher [^18^F]MK6240 SUVRs in Braak staging I–IV ROIs as compared to *APOE*ε4 non-carriers. *ADNI: Male:* No significant difference in [^18^F]flortaucipir SUVRs was observed between *APOE*ε4 carriers and *APOE*ε4 non-carriers. *Female: APOE*ε4 carriers with MCI showed significantly higher [^18^F]flortaucipir SUVRs in Braak staging I–VI ROIs as compared to *APOE*ε4 non-carriers. *APOE*ε4 carriers with Alzheimer’s disease also showed significantly higher tau levels in Braak staging III–IV ROIs. ****P < 0.001, ***P < 0.005, **P < 0.01, *P < 0.05. Independent samples *t*-tests were performed for comparisons between *APOE*ε4 carriers and *APOE*ε4 non-carriers with different clinical diagnosis regarding their tau burden in different ROIs.

We first perform ROI analyses to examine how *APOE* modulated the tau burden in male and female subjects, respectively. In the TRIAD cohort, the tau level of male *APOE*ε4 carriers did not differ from *APOE*ε4 non-carriers. In contrast, female *APOE*ε4 carriers showed significantly higher tau burden in Braak staging I–IV ROIs^45^ in comparison to the non-carriers (Fig. 2). Consistent with the findings in the TRIAD cohort, in the ADNI cohort, *APOE* did not exert a significant effect on tau level in male. Female with ε4 allele(s) showed significantly higher tau burden in Braak staging I–IV ROIs as compared to the non-carriers (Fig. 2). To ensure that the results were not driven by an effect of the clinical status, the subjects were stratified by their clinical diagnosis. As shown in Fig. 3, no significant difference in the tau burden was observed between the male *APOE*ε4 carriers and the *APOE*ε4 non-carriers in any of the diagnostic groups. In contrast, tau-PET SUVRs were found to be significantly higher in the female *APOE*ε4 carriers in comparison to the non-carriers with the same clinical diagnosis. In the TRIAD cohort, both cognitively unimpaired and cognitively impaired female *APOE*ε4 carriers showed significantly higher tau burden in Braak staging I–IV ROIs as compared to the non-carriers (Fig. 3). Whereas in the ADNI cohort, for women with a diagnosis of MCI, tau burden in Braak staging I–IV ROIs was significantly higher in the *APOE*ε4 carriers in comparison to the non-carriers (Fig. 3). Linear regression analyses were also performed to investigate the relationships between *APOE* and tau-PET SUVRs in two sexes (Supplementary Tables 1 and 2).

### Sex-specific vulnerability to tau in female *APOEε4* carriers

**Figure 4:**
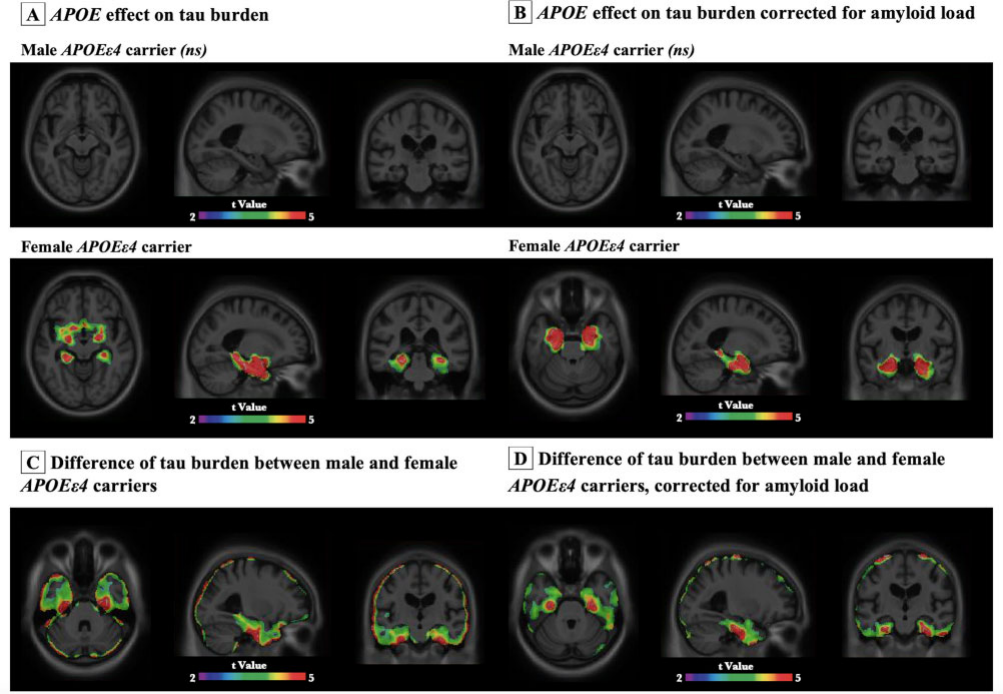
***APOE*ε4 was associated with higher tau load in medial temporal regions in women but not in men.** Images represent voxel-based *t*-statistical parametric maps (corrected for multiple comparisons; cluster threshold of *P* < 0.001) overlaid on the structural MRI ADNI reference template. Age, educational attainment and clinical diagnosis were used as covariates in the model. (**A**) No significant effect of *APOE* on tau burden was observed in male. Female *APOE*ε4 carriers showed significantly higher [^18^F]MK6240 SUVRs in hippocampus, entorhinal and parahippocampal cortices as compared to female *APOE*ε4 non-carriers. (**B**) After accounting for the amyloid load, tau burden remained no difference between male *APOE*ε4 carriers and *APOE*ε4 non-carriers. Higher tau burden was again found in female *APOE*ε4 carriers in hippocampus, entorhinal and parahippocampal cortices in comparison to female *APOE*ε4 non-carriers. (**C and D**) Female *APOE*ε4 carriers showed significantly higher tau burden in medial temporal regions as compared to male *APOE*ε4 carriers (results remained significant after corrected for amyloid load).

We next performed voxelwise analyses to study how *APOE* modulates regional vulnerability to tau accumulation in male and female. Voxelwise analyses revealed no significant difference in tau burden between male *APOEε4* carriers and the non-carriers. In contrast, female *APOEε4* carriers showed significantly higher tau burden in Braak staging I–II ROIs including hippocampus, entorhinal and parahippocampal cortices as compared to the *APOEε4* non-carriers (Fig. 4A). To ensure the results were not driven by the amyloid, we corrected for the neocortical amyloid load (Fig. 4B). We further compared the tau burden between male and female *APOEε4* carriers. As shown in Fig. 4C and D, results from voxelwise analyses indicated that female *APOEε4* carriers had significantly higher tau burden in the medial temporal structures including the hippocampus, entorhinal and parahippocampal cortices as compared to their male counterparts, after correcting for amyloid load. Age, educational attainment and clinical diagnosis were used as covariates in all the models.

### Interactive but not independent effect of *APOE* and sex potentiates early tau deposition

**Figure 5:**
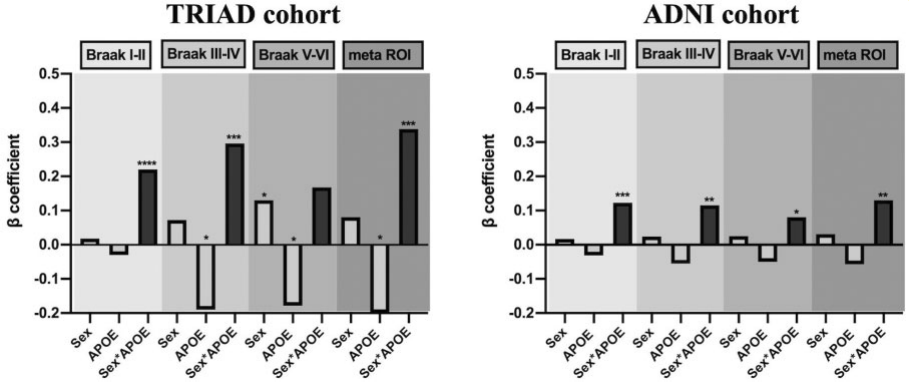
**Interactive rather than the main effect of *APOE* and sex modulated tau load.** This figure illustrates the beta-coefficients of three variables of interest in the multivariate linear regression analyses (the statistical details can be found in Table 2). The regression models were performed in 277 individuals from the TRIAD cohort, and 221 individuals from the ADNI cohort separately. *TRIAD: APOE-*by-sex interactive effect was significantly associated with tau burden in Braak staging I–IV ROIs and meta ROIs. *ADNI: APOE-*by-sex interactive effect was significantly associated with tau burden in Braak staging I–VI ROIs and meta ROIs. Age, educational attainment, clinical diagnosis and amyloid load were used as covariates in the models. ****P < 0.001, ***P < 0.005, **P < 0.01, *P < 0.05.

**Figure 6:**
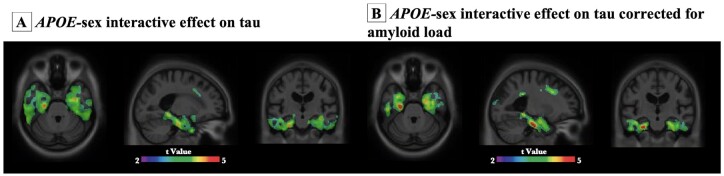
**Voxel-based interaction analyses revealed that *APOE*-by-sex interactive effect was associated with tau load in medial temporal regions.** *APOE-*by-sex interactive effect was significantly associated with tau load in medial temporal regions. Images represent voxel-based *t*-statistical parametric maps (corrected for multiple comparisons; cluster threshold of *P* < 0.001) overlaid on the structural MRI ADNI reference template. Age, educational attainment and clinical diagnosis were used as covariates in the models. Analyses performed with and without correction for amyloid were presented in **B** and **A**, respectively.

Finally, multivariate linear regression models were performed to assess the main effect and the interactive effect of *APOE* and sex on tau burden. Detailed statistic is listed in Table 2. As shown in Fig. 5, in both TRIAD and ADNI cohort, the *APOE-*by-sex interactive effect was positively associated with tau burden in the Braak staging I–IV ROIs as well as the meta ROIs. Voxelwise analyses were also conducted and it showed that the *APOE*-by-sex interactive effect determined tau deposition in the medial temporal regions (Fig. 6A). The results remained significant after correcting for the neocortical amyloid load (Fig. 6B). It is important to note that this *APOE-*by*-*sex interactive effect only showed a positive association with tau burden, but not with Aβ load (Supplementary Table 3).

**Table 2:** Regression analysis revealed *APOE-*sex interactive effect was associated with tau-PET SUVRs

TRIAD								
	Braak I–II				Braak III–IV			
	Estimate	SE	*t*-value	*P-*value	Estimate	SE	*t*-value	*P-*value
Age	−0.0021	0.0017	−1.253	0.211	−0.0112	0.0028	−4.069	****<0.001
Female	**0.0187**	**0.0363**	**0.514**	**0.607**	**0.0721**	**0.0600**	**1.201**	**0.231**
*APOEε4*	−**0.0307**	**0.0458**	−**0.670**	**0.503**	−**0.1906**	**0.0755**	−**2.525**	*0.012
Education	0.0028	0.0041	0.696	0.487	0.0045	0.0068	0.664	0.507
Clinical Status								
MCI	0.2761	0.0965	2.86	***0.004	0.5912	0.1592	3.713	****<0.001
AD	0.5557	0.0949	5.852	****<0.001	1.6191	0.1566	10.34	****<0.001
Global Amyloid	0.4076	0.0385	10.59	****<0.001	0.4074	0.0635	6.417	****<0.001
Female^*^APOEε4	**0.2235**	**0.0596**	**3.752**	****<0.001	**0.2965**	**0.0982**	**3.018**	***0.003
	**Braak V–VI**				**meta ROI**			
	**Estimate**	**SE**	***t*-value**	***P-*value**	**Estimate**	**SE**	***t*-value**	***P-*value**
Age	−0.0149	0.0026	−5.706	****<0.001	−0.0116	0.0030	−3.921	****<0.001
Female	**0.1341**	**0.0567**	**2.365**	*0.019	**0.0819**	**0.0644**	**1.273**	**0.204**
*APOEε4*	−**0.1778**	**0.0713**	−**2.493**	*0.013	−**0.2050**	**0.0810**	−**2.532**	*0.012
Education	0.0074	0.0064	1.155	0.249	0.0026	0.0072	0.361	0.718
Clinical Status								
MCI	0.6444	0.1504	4.283	****<0.001	0.6261	0.1708	3.665	****<0.001
AD	1.5426	0.1480	10.42	****<0.001	1.696	0.1680	10.095	****<0.001
Global Amyloid	0.2755	0.0600	4.593	****<0.001	0.4959	0.0681	7.281	****<0.001
Female^*^APOEε4	**0.1683**	**0.0928**	**1.813**	**0.071**	**0.3386**	**0.1054**	**3.212**	***0.0015
**ADNI**								
	**Braak I**−**II**				**Braak III**−**IV**			
	**Estimate**	**SE**	***t*-value**	***P-*value**	**Estimate**	**SE**	***t*-value**	***P-*value**
Age	0.0005	0.0015	0.32	0.746	−0.0044	0.0017	−2.62	**0.009
Female	**0.0169**	**0.0247**	**0.68**	**0.494**	**0.0239**	**0.0267**	**0.89**	**0.37**
*APOEε4*	−**0.031**	**0.029**	−**1.06**	**0.289**	−**0.055**	**0.032**	−**1.73**	**0.084**
Education	<0.001	0.0038	−0.019	0.984	0.0019	0.0041	0.476	0.634
Clinical status MCI	0.061	0.022	2.77	**0.006	0.066	0.024	2.79	***0.005
AD	0.156	0.036	4.33	****<0.001	0.327	0.039	8.349	****<0.001
Global amyloid	0.146	0.041	3.51	****<0.001	0.211	0.045	4.69	****<0.001
Female^*^APOEε4	**0.123**	**0.041**	**2.97**	***0.003	**0.115**	**0.045**	**2.58**	**0.010
	**Braak V**−**VI**				**meta ROI**			
	**Estimate**	**SE**	***t*-value**	***P-*value**	**Estimate**	**SE**	***t*-value**	***P-*value**
Age	−0.005	0.0014	−3.468	****<0.001	−0.0038	0.0018	−2.059	*0.04
Female	**0.025**	**0.0227**	**1.098**	**0.273**	**0.0309**	**0.0293**	**1.054**	**0.293**
*APOEε4*	−**0.05**	**0.027**	−**1.83**	**0.068**	−**0.057**	**0.035**	−**1.638**	**0.102**
Education	0.0018	0.0034	0.532	0.595	0.002	0.0045	0.571	0.568
Clinical status MCI	0.049	0.020	2.42	*0.016	0.073	0.026	2.81	***0.005
AD	0.216	0.033	6.49	****<0.001	0.370	0.043	8.608	****<0.001
Global amyloid	0.174	0.038	4.54	****<0.001	0.249	0.049	5.048	****<0.001
Female^*^APOEε4	**0.08**	**0.038**	**2.09**	*0.037	**0.130**	**0.049**	**2.641**	**0.009

In both TRIAD and ADNI cohort, the *APOE-*sex interaction effect was observed to be associated with tau burden. Age, educational attainment, clinical diagnosis and neocortical Aβ SUVR were used as covariates in the model. The values marked bold are from variables that we would want to readers to focus on. If no * is marked, then the p-value is not significant.

**** *P *<* *0.001,

*** *P *<* *0.005,

** *P *<* *0.01,

* *P *<* *0.05.

## Discussion

In this study, we provide evidence that *APOEε4* carriage status is highly associated with early tau load in females, but not in males. In addition, it was the interaction between *APOE* and sex, rather than their independent main effects that potentiated early tau deposition. These findings are consistent with our hypotheses and extend prior findings^17^ by showing that the *APOE*-by-sex interaction in tau accumulation is present even after co-varying out the effects of age, education and clinical diagnosis.

A previous post-mortem study showed that brains of Alzheimer’s disease patients carrying two *APOEε4* alleles have more tau aggregates than those carrying either one or no *APOEε4* alleles.^46^ We also have shown a dose-dependent effect of *APOE* on tau load.^36^ Of note, our findings indicate that this might be highly driven by female subjects since we did not observe an effect of *APOE* on tau load in male subjects (Figs 2 and 3). Interestingly, one recent study implicates that a novel locus on chromosome 7 (rs34331204) confers male-specific protection from tau pathology,^47^ which emphasizes the value to elucidate sex differences in the genetic architecture of Alzheimer’s disease. In addition, a previous study reported a stronger association between *APOEε4* and higher CSF tau burden among women compared with men, yet this association was only observed in individuals with evident Aβ pathology.^16^ Although a substantial amount of studies suggests that Aβ pathology drives neurofibrillary tangles in Alzheimer’s disease, emerging preclinical evidence indicates that tau pathology can progress independently of Aβ accumulation, and arises downstream of genetic risk factors for Alzheimer’s disease.^48^^,^^49^ In order to elucidate this point, further analyses were conducted specifically in 117 subjects from the TRIAD cohort and 153 subjects from the ADNI cohort with Aβ positivity on amyloid-PET. Aβ positivity was defined as SUVR above a cut-off value of 1.55 for AZD4694 PET,^43^ and 1.11 for AV45 PET.^50^^,^^51^ We evaluated the relationship between *APOE*-by-sex interactive effect and tau burden, with and without taking amyloid as a covariate. The relationship between *APOE*-by-sex interactive effect and amyloid load, as well as the relationship between amyloid-PET SUVR and tau-PET SUVR, was also analysed (Supplementary Table 4). Consistent with previous findings, we found that in the TRIAD cohort, both global amyloid load and *APOE*-by-sex interactive effect were significantly associated with tau-PET SUVR in Braak I–IV ROIs. Whereas in the late Braak ROIs (Braak V–VI), the only amyloid load was associated with tau burden. Furthermore, as shown in the mediation analysis (Supplementary Table 5), there was a direct effect between *APOE*-by-sex interaction and tau-PET SUVRs. On the contrary, in the ADNI cohort, the amyloid load seems to be the most important factor to affect tau burden in amyloid-positive subjects. This, in fact, is in line with results from this study, as we found that *APOE*-by-sex interaction affects Alzheimer’s disease patients in a relatively early disease stage (Braak I–II). Thus with patients in the ADNI cohort being averagely older and relatively advanced in the disease stage, it is not surprised to see that *APOE*-by-sex interactive effect was not strongly associated with the tau burden in Aβ-positive patients. With these, we concluded that the *APOEε4*-imposed abnormal tau accumulation was associated with, yet not dependent of Aβ load, and future studies designed to understand the mechanisms by which *APOE* contributes to or counteracts Alzheimer’s disease pathogenesis via Aβ-dependent and Aβ-independent pathways is critical.

One mechanism that could underlie this sex difference of *APOE* effect on tau is the sex difference in the lifetime exposures to sex hormones, particularly 17β-oestradiol levels.^52^^,^^53^ Evidence from animal models suggests that oestradiol seems to protect against tau hyperphosphorylation, particularly among female rats,^54^ and oestrogen receptor α co-localizes with neurofibrillary tangles. Interestingly, the α receptor also appears to be responsible for the oestrogen-mediated upregulation of *APOE* expression,^55^ indicating a possible mechanism in which oestrogen and *APOE* act synergistically in postmenopausal women. In support of this, Bove and colleagues^56^ reported that early surgical menopause doubled the risk of dementia, and was associated with late-onset Alzheimer’s disease pathology as well as cognitive decline. In addition, loss of oestradiol has also been implied to cause hippocampal dysfunction.^57^ Thus, experimental approaches are needed to better understand the potential contribution of sex hormone differences between men and women in driving the observed sex differences of *APOE* effect on tau load.

In this study, we leveraged tau-PET imaging data from two independent cohorts and showed that female *APOEε4* carriers are more susceptible to the accumulation of tau. This is in line with one recent finding that indicates sex modulates *APOE* effect on tau deposition in MCI patients.^58^ Importantly, we further provide evidence that this sex difference of *APOE* effect on tau burden remained significant after corrected for amyloid load, and it is the interaction between *APOE* and sex potentiates tau burden, rather than their independent effects. In addition, this *APOE-*by-sex interactive effect is more evident in medial temporal regions known to succumb to Alzheimer’s disease early. Our findings highlight the importance of sex difference in Alzheimer’s disease research, and the exclusion of sex as a variable has impeded faster advancement in the detection, treatment and care of Alzheimer’s disease across the clinical spectrum.^19^ Indeed, sex-specific genetic associations have important implication for clinical intervention. In several clinical studies, *APOEε4* carriers and non-carriers responded differently to the treatment. Of one example, in a phase III trial of bapineuzumab, a humanized anti-Aβ monoclonal antibody, *APOEε4* status was associated with differences in both Aβ and tau load in mild-to-moderate Alzheimer’s disease patients.^59^ In addition, it has also been reported that the *APOE-*by-sex interactions influence the efficacy of intranasal insulin treatments.^60^ The interface of sex and *APOEε4* thus represents a unique nexus to take the first step towards precision medicine approaches. This could facilitate the development of different Alzheimer’s disease treatment strategies and improve outcomes for both sexes.

One strength of this study is the fact that extensive analyses including explorations into age, educational attainment, diagnostic status and neocortical Aβ load were performed in a large sample size. Importantly, the replication of results obtained with first-generation and second-generation tau-PET ligands in two independent cohorts is a methodological advance. However, limitations should be considered when interpreting our results. First, the cross-sectional nature of this study limits our ability to make causal inferences. The previous finding from a longitudinal study indicates that among MCI due to Alzheimer’s disease subjects, the mean worsening in the cognitive score is significantly greater in women than in men.^30^ In this study, although a significantly higher tau burden was observed in women, no significant difference in Mini-Mental State Examination score between the two sexes was found. Whether women are more susceptible^17^ or more resilient^61^ to tau remains unanswered. Longitudinal study will be needed to evaluate if interaction between *APOEε4* and sex facilitates tau accumulation and spreading in women compared with men, and if this accelerates the worsening in cognitive function. Secondly, it is important to highlight that this study is phenomenological and does not intent to investigate the mechanisms underlying *APOE-*by-sex interactive effect on tau load. Future works are needed to determine whether this interactive effect results in increased phosphorylation^62^ or cortical spreading of tau in a sex-specific manner as well. In addition, the possible associations between the *APOE-*by-sex interaction, Aβ load,^63^ microglial activation,^64^ synaptic function and neurodegeneration in relation to tau burden also remain to be investigated. Finally, both TRIAD and ADNI cohort are relatively homogeneous across race and ethnicity. Thus, it is crucial to replicate and extend findings from this study in other independent cohorts with greater diversity to know if these findings can be generalized to other racial and ethnic groups.

In conclusion, results from our study demonstrate that *APOE* modulates sex-specific regional vulnerability for tau, and the interaction between *APOE* and sex potentiates early tau deposition. This has important clinical implications. In clinical trials targeting tau, both *APOEε4* carriage and sex are of critical importance for identifying individuals with the highest probability to develop tau pathology. In addition, the dosage of anti-tau treatment should be adjusted by the *APOE-*by-sex group. Furthermore, sex difference needs to be taken into consideration in studies exploring tau-dependent mechanisms underlying *APOEε4*-mediated Alzheimer’s disease risk. Ultimately, an increased focus on sex as a biological variable in Alzheimer’s disease genetic architecture, and the inclusion of sex difference in Alzheimer’s disease analytical models will advance the screening, prognosis, treatment and care of Alzheimer’s disease across the clinical spectrum.

## Supplementary material

Supplementary material is available at *Brain Communications* online.

## Supplementary Material

fcab126_Supplementary_Data

## Abbreviations

ADNI =: Alzheimer’s Disease Neuroimaging Initiative
*APOE* =: Apolipoprotein E
CDR =: Clinical Dementia Rating
MCI =: mild cognitive impairment
ROI =: region of interest
SUVR =: standardized uptake value ratio
TRIAD: = Translational Biomarkers in Aging and Dementia
