# Supplementary material for: Interactive rather than independent effect of APOE and sex potentiates tau deposition in women
Source: Brain Commun. 2021 Jun 7;3(2):fcab126. doi: 10.1093/braincomms/fcab126 (PMC8226193; doi:10.1093/braincomms/fcab126)
Supplement: fcab126_Supplementary_Data [file fcab126_supplementary_data.docx]

| **Supplementary Table 1. Association between *APOE* and Tau-PET SUVR in TRIAD cohort** | | | | | | | | |
| --- | --- | --- | --- | --- | --- | --- | --- | --- |
| **TRIAD Women** | | | | | | | | |
|  | **Braak I-II** | | | | **Braak III-IV** | | | |
|  | **Estimate** | **SE** | ***t* Value** | ***P* Value** | **Estimate** | **SE** | ***t* Value** | ***P* Value** |
| **Age** | 0.0007 | 0.0013 | 0.574 | 0.567 | -0.0025 | 0.0019 | -1.29 | 0.199 |
| **Education** | -0.0012 | 0.0063 | -0.199 | 0.842 | 0.0017 | 0.0092 | 0.18 | 0.856 |
| **Clinical Status**  **AD** | 0.719 | 0.086 | 8.315 | **^****^**<0.001 | 1.402 | 0.126 | 11.128 | **^****^**<0.001 |
| **MCI** | 0.167 | 0.064 | 2.626 | **^**^**0.009 | 0.182 | 0.093 | 1.960 | 0.051 |
| ***APOEε4*** | **0.213** | **0.047** | **4.518** | ******<0.001** | **0.189** | **0.069** | **2.745** | ****0.0067** |
| **Global Amyloid** | 0.321 | 0.052 | 6.121 | **^****^**<0.001 | 0.299 | 0.076 | 3.914 | **^****^**<0.001 |
|  | **Braak V-VI** | | | | **meta ROI** | | | |
|  | **Estimate** | **SE** | ***t* Value** | ***P* Value** | **Estimate** | **SE** | ***t* Value** | ***P* Value** |
| **Age** | -0.0029 | 0.0019 | -1.522 | 0.130 | -0.0026 | 0.0021 | -1.26 | 0.209 |
| **Education** | 0.0079 | 0.0091 | 0.868 | 0.386 | -0.0009 | 0.0099 | -0.088 | 0.930 |
| **Clinical Status**  **AD** | 1.301 | 0.124 | 10.49 | **^****^**<0.001 | 1.532 | 0.136 | 11.297 | **^****^**<0.001 |
| **MCI** | 0.149 | 0.091 | 1.636 | 0.104 | 0.240 | 0.100 | 2.402 | **^*^**0.017 |
| ***APOEε4*** | **0.142** | **0.068** | **2.098** | ***0.037** | **0.199** | **0.074** | **2.691** | ****0.008** |
| **Global Amyloid** | 0.111 | 0.075 | 1.478 | 0.141 | 0.382 | 0.082 | 4.642 | **^****^**<0.001 |
| **TRIAD Men** | | | | | | | | |
|  | **Braak I-II** | | | | **Braak III-IV** | | | |
|  | **Estimate** | **SE** | ***t* Value** | ***P* Value** | **Estimate** | **SE** | ***t* Value** | ***P* Value** |
| **Age** | 0.0001 | 0.0022 | 0.061 | 0.952 | -0.0021 | 0.0038 | -0.551 | 0.582 |
| **Education** | 0.0143 | 0.0089 | 1.606 | 0.111 | 0.0233 | 0.015 | 1.554 | 0.123 |
| **Clinical Status**  **AD** | 0.405 | 0.127 | 3.177 | **^***^**0.002 | 1.298 | 0.214 | 6.054 | **^****^**<0.001 |
| **MCI** | 0.016 | 0.090 | 0.182 | 0.856 | 0.025 | 0.152 | 0.165 | 0.869 |
| ***APOEε4*** | -0.035 | 0.071 | -0.491 | 0.624 | -0.179 | 0.120 | -1.488 | 0.139 |
| **Global Amyloid** | 0.550 | 0.073 | 7.491 | **^****^**<0.001 | 0.456 | 0.123 | 3.689 | **^****^**<0.001 |
|  | **Braak V-VI** | | | | **meta ROI** | | | |
|  | **Estimate** | **SE** | ***t* Value** | ***P* Value** | **Estimate** | **SE** | ***t* Value** | ***P* Value** |
| **Age** | -0.0069 | 0.003 | -2.271 | 0.025 | -0.001 | 0.0043 | -0.228 | 0.820 |
| **Education** | 0.0091 | 0.012 | 0.756 | 0.451 | 0.0358 | 0.0172 | 2.083 | 0.039 |
| **Clinical Status**  **AD** | 1.057 | 0.172 | 6.152 | **^****^**<0.001 | 1.291 | 0.246 | 5.243 | **^****^**<0.001 |
| **MCI** | 0.062 | 0.122 | 0.509 | 0.611 | -0.0033 | 0.175 | -0.019 | 0.985 |
| ***APOEε4*** | -0.061 | 0.096 | -0.631 | 0.529 | -0.203 | 0.138 | -1.472 | 0.144 |
| **Global Amyloid** | 0.314 | 0.099 | 3.173 | **^***^**0.002 | 0.538 | 0.142 | 3.794 | **^****^**<0.001 |
| In male participants, no significant association between *APOEε4* carriage status and tau load was observed. In women, *APOEε4* carriage status showed significant correlation with [^18^F]MK6240 SUVR in Braak I-II, III-IV, V-VI ROIs as well as meta ROIs, after correcting for age, education level, clinical diagnosis and neocortical Aβ level. Braak stage I-II: transentorhinal areas; Braak stage III-IV: limbic regions; Braak stage V-VI: isocortical association areas. Meta-ROI region includes entorhinal, amygdala, parahippocampal, fusiform, inferior temporal, and middle temporal. Cerebellar grey matter was used as reference region. ^****^*P* < 0.001, ^***^*P* < 0.005, ^**^*P* < 0.01, ^*^*P* < 0.05. | | | | | | | | |

| **Supplementary Table 2. Association between *APOE* and Tau-PET SUVR in ADNI cohort** | | | | | | | | |
| --- | --- | --- | --- | --- | --- | --- | --- | --- |
| **ADNI Women** | | | | | | | | |
|  | **Braak I-II** | | | | **Braak III-IV** | | | |
|  | **Estimate** | **SE** | ***t* Value** | ***P* Value** | **Estimate** | **SE** | ***t* Value** | ***P* Value** |
| **Age** | -0.0012 | 0.002 | -0.617 | 0.538 | -0.0053 | 0.0023 | -2.273 | **^*^**0.025 |
| **Education** | 0.0016 | 0.0047 | 0.333 | 0.739 | 0.0089 | 0.0055 | 1.607 | 0.110 |
| **Clinical Status**  **AD** | 0.257 | 0.046 | 5.612 | **^****^**<0.001 | 0.445 | 0.053 | 8.336 | **^****^**<0.001 |
| **MCI** | 0.065 | 0.029 | 2.187 | **^*^**0.03 | 0.074 | 0.034 | 2.157 | **^*^**0.033 |
| ***APOEε4*** | **0.076** | **0.027** | **2.825** | *****0.005** | 0.049 | 0.031 | 1.560 | 0.121 |
| **Global Amyloid** | 0.174 | 0.057 | 3.025 | **^***^**0.003 | 0.209 | 0.067 | 3.129 | **^***^**0.002 |
|  | **Braak V-VI** | | | | **meta ROI** | | | |
|  | **Estimate** | **SE** | ***t* Value** | ***P* Value** | **Estimate** | **SE** | ***t* Value** | ***P* Value** |
| **Age** | -0.0049 | 0.0018 | -2.653 | **^**^**0.0090 | -0.0045 | 0.0025 | -1.790 | 0.076 |
| **Education** | 0.0085 | 0.0044 | 1.908 | 0.06 | 0.0094 | 0.0060 | 1.577 | 0.117 |
| **Clinical Status**  **AD** | 0.321 | 0.043 | 7.511 | **^****^**<0.001 | 0.486 | 0.058 | 8.434 | **^****^**<0.001 |
| **MCI** | 0.046 | 0.027 | 1.682 | 0.095 | 0.083 | 0.037 | 2.229 | **^*^**0.027 |
| ***APOEε4*** | 0.026 | 0.025 | 1.024 | 0.308 | 0.061 | 0.034 | 1.811 | 0.072 |
| **Global Amyloid** | 0.146 | 0.053 | 2.728 | **^**^**0.007 | 0.253 | 0.072 | 3.510 | **^****^**<0.001 |
| **ADNI Men** | | | | | | | | |
|  | **Braak I-II** | | | | **Braak III-IV** | | | |
|  | **Estimate** | **SE** | ***t* Value** | ***P* Value** | **Estimate** | **SE** | ***t* Value** | ***P* Value** |
| **Age** | 0.0024 | 0.0023 | 1.039 | 0.30 | -0.0028 | 0.0024 | -1.161 | 0.247 |
| **Education** | -0.0006 | 0.0059 | -0.111 | 0.912 | -0.0035 | 0.0061 | -0.586 | 0.558 |
| **Clinical Status**  **AD** | 0.052 | 0.055 | 0.943 | 0.347 | 0.221 | 0.057 | 3.873 | **^****^**<0.001 |
| **MCI** | 0.056 | 0.032 | 1.743 | 0.084 | 0.063 | 0.033 | 1.925 | 0.056 |
| ***APOEε4*** | -0.020 | 0.032 | -0.627 | 0.532 | -0.047 | 0.033 | -1.404 | 0.163 |
| **Global Amyloid** | 0.116 | 0.059 | 1.964 | 0.052 | 0.193 | 0.061 | 3.184 | **^***^**0.002 |
|  | **Braak V-VI** | | | | **meta ROI** | | | |
|  | **Estimate** | **SE** | ***t* Value** | ***P* Value** | **Estimate** | **SE** | ***t* Value** | ***P* Value** |
| **Age** | -0.0042 | 0.0021 | -1.964 | 0.052 | -0.0024 | 0.0027 | -0.883 | 0.378 |
| **Education** | -0.0034 | 0.0054 | -0.637 | 0.525 | -0.0028 | 0.0068 | -0.407 | 0.684 |
| **Clinical Status**  **AD** | 0.128 | 0.051 | 2.527 | **^*^**0.013 | 0.266 | 0.064 | 4.148 | **^****^**<0.001 |
| **MCI** | 0.053 | 0.029 | 1.826 | 0.070 | 0.069 | 0.037 | 1.881 | 0.062 |
| ***APOEε4*** | -0.045 | 0.030 | -1.505 | 0.135 | -0.049 | 0.037 | -1.301 | 0.195 |
| **Global Amyloid** | 0.174 | 0.054 | 3.224 | **^***^**0.002 | 0.228 | 0.068 | 3.355 | **^****^**0.001 |
| In male participants, no significant association between *APOEε4* carriage status and tau load was observed. In women, *APOEε4* carriage status showed significant correlation with [^18^F]flortaucipir SUVR in Braak I-II ROIs, after correcting for age, education level, clinical diagnosis and neocortical Aβ level. Braak stage I-II: transentorhinal areas; Braak stage III-IV: limbic regions; Braak stage V-VI: isocortical association areas. Meta-ROI region includes entorhinal, amygdala, parahippocampal, fusiform, inferior temporal, and middle temporal. Cerebellar grey matter was used as reference region. ^****^*P* < 0.001, ^***^*P* < 0.005, ^**^*P* < 0.01, ^*^*P* < 0.05. | | | | | | | | |

| **Supplementary Table 3. Interactive effect of *APOE* and sex on Aβ load** | | | | | | | | |
| --- | --- | --- | --- | --- | --- | --- | --- | --- |
|  | **TRIAD** | | | | **ADNI** | | | |
|  |  |  |  |  |  |  |  |  |
|  | **Estimate** | **SE** | ***t* Value** | ***P* Value** | **Estimate** | **SE** | ***t* Value** | ***P* Value** |
| **Age** | 0.0088 | 0.0016 | 5.62 | **^****^**<0.001 | 0.0068 | 0.0023 | 2.93 | **^***^**0.0037 |
| **Female** | -0.0003 | 0.070 | -0.0039 | 0.997 | -0.0251 | 0.037 | -0.679 | 0.50 |
| **Education** | 0.0036 | 0.0072 | 0.504 | 0.61 | -0.0027 | 0.0056 | -0.481 | 0.63 |
| **Clinical Status**  **AD** | 0.942 | 0.085 | 11.1 | **^****^**<0.001 | 0.190 | 0.053 | 3.588 | **^****^**<0.001 |
| **MCI** | 0.455 | 0.069 | 6.6 | **^****^**<0.001 | 0.029 | 0.033 | 0.891 | 0.37 |
| ***APOEε4*** | -0.066 | 0.0847 | -0.776 | 0.438 | **0.094** | **0.044** | **2.136** | ***0.03** |
| **Female* *APOEε4*** | 0.175 | 0.111 | 1.584 | 0.11 | 0.030 | 0.062 | 0.486 | 0.63 |
| In both TRIAD and ADNI cohort, no significant *APOE-*sex interactive effect on Aβ level was observed. Age, educational attainment and clinical diagnosis were used as covariates in the model. ^****^*P* < 0.001, ^***^*P* < 0.005, ^**^*P* < 0.01, ^*^*P* < 0.05. | | | | | | | | |

| **Supplementary Table 4.** | 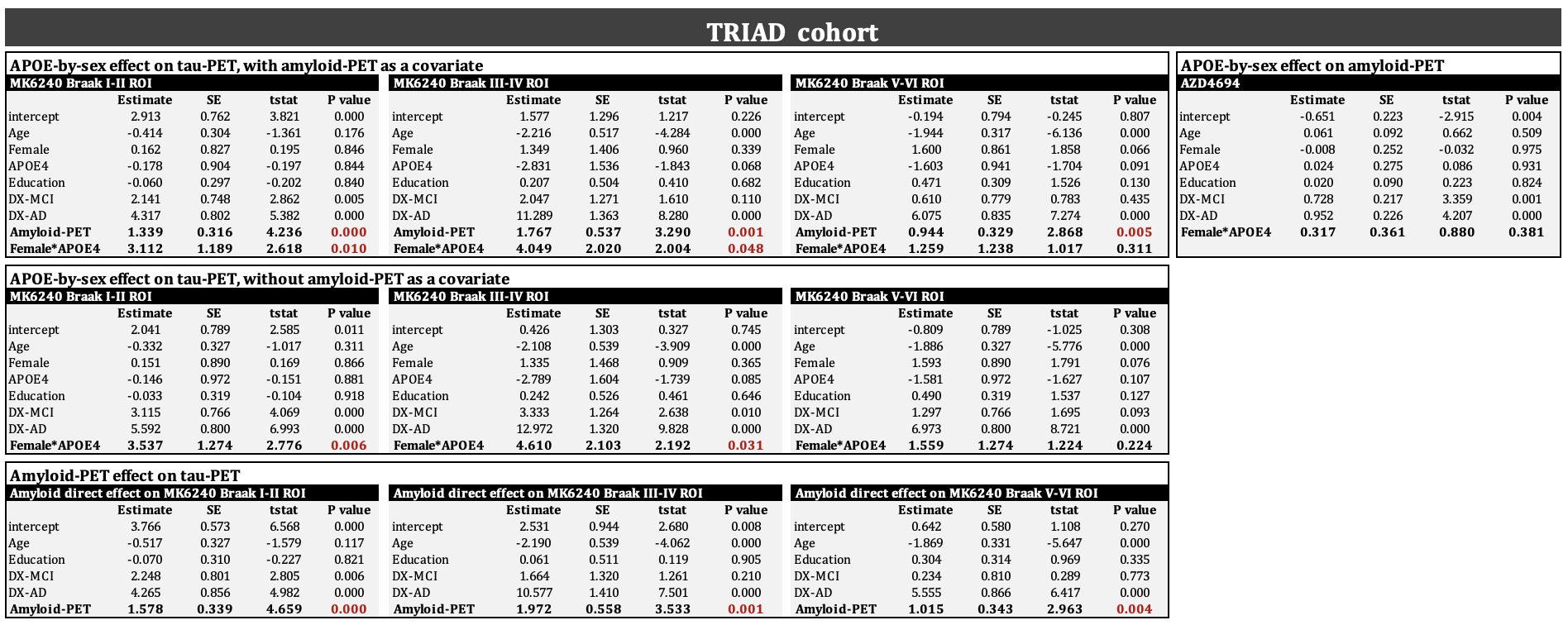 |
| --- | --- |

| **Supplementary Table 4 *(continued)*** | 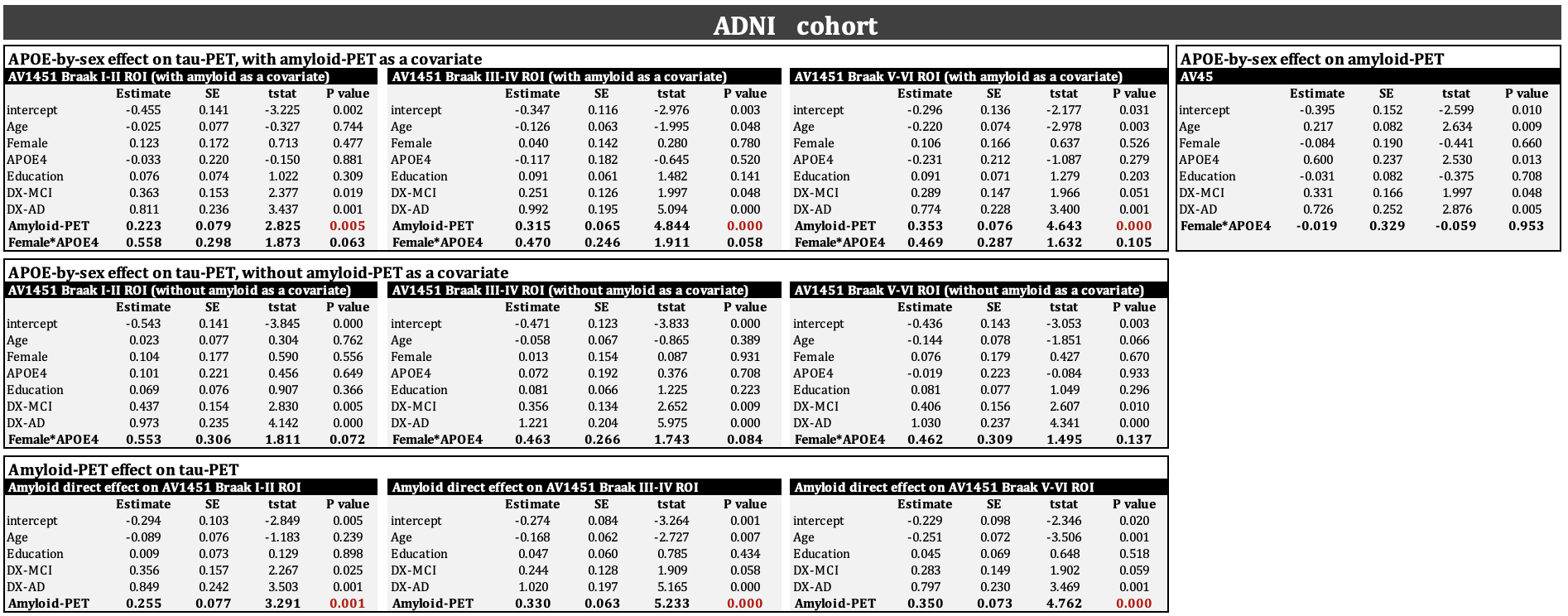 |
| --- | --- |

| **Supplementary Table 5. Mediation Analysis** | **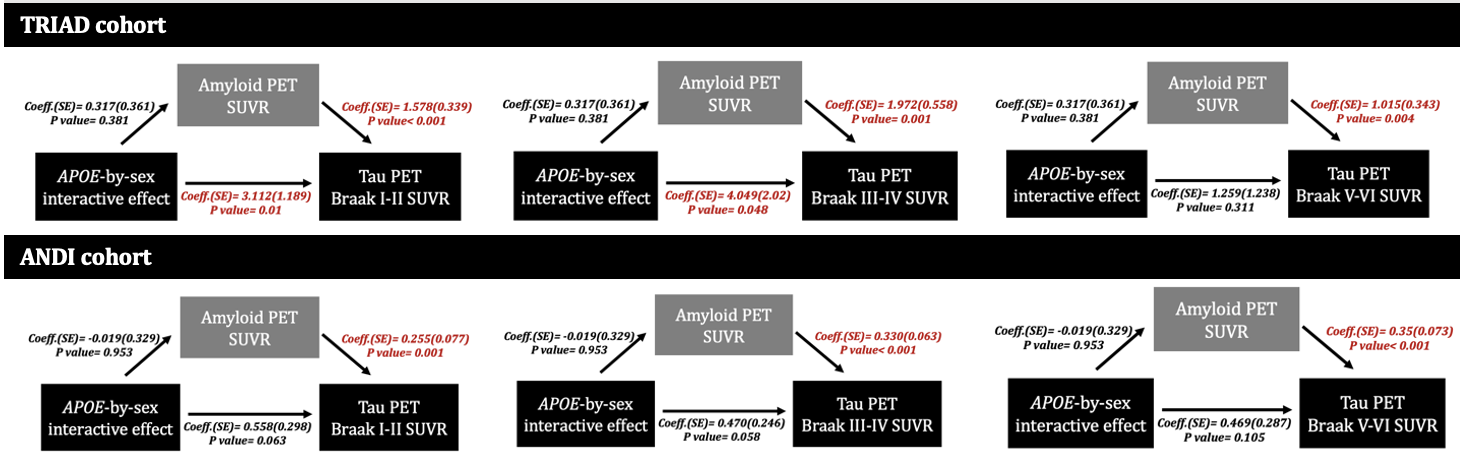** |
| --- | --- |
